# Healthcare worker perspectives on mother’s insufficient milk supply in Malawi

**DOI:** 10.1186/s13006-022-00460-1

**Published:** 2022-02-23

**Authors:** Olivia Piccolo, Mai-Lei Woo Kinshella, Sangwani Salimu, Marianne Vidler, Mwai Banda, Queen Dube, Kondwani Kawaza, David M. Goldfarb, Alinane Linda Nyondo-Mipando

**Affiliations:** 1 Department of Health Sciences, McMaster University, Hamilton, Canada; 2 Department of Obstetrics and Gynaecology, BC Children’s and Women’s Hospital and University of British Columbia, Vancouver, Canada; 3 Department of Pathology and Laboratory Medicine, BC Children’s and Women’s Hospitals and University of British Columbia, Vancouver, Canada; 4 Department of Pediatrics and Child Health, College of Medicine, University of Malawi, Blantyre, Malawi; 5 Department of Pediatrics, Queen Elizabeth Central Hospital, Blantyre, Malawi; 6 Department of Health Systems and Policy, College of Medicine, University of Malawi, Blantyre, Malawi

**Keywords:** Breastfeeding, Breastmilk insufficiency, Perceived insufficient milk supply, Low- and middle-income country, Health care worker perspectives

## Abstract

**Background:**

Human milk insufficiency is a significant barrier to implementing breastfeeding, and it is identified as a prevalent concern in 60–90% of mothers in low-and-middle-income countries. Breastmilk insufficiency can lead to hypoglycemia, hypernatremia, nutritional deficiencies, and failure to thrive in newborns and infants. Studies investigating the impact of breastfeeding interventions to improve milk production highlight inconsistencies between healthcare workers and mothers perceived support, as well as gaps in practical knowledge and training. The aim of this study was to determine perceptions surrounding human milk insufficiency from Malawian healthcare workers.

**Methods:**

This study is a secondary analysis of 39 interviews with healthcare workers from one tertiary and three district hospitals in Malawi employing content analysis. Interviewed healthcare workers included nurses, clinical officers, midwives, and medical doctors. An inclusive coding framework was developed to identify themes related to human milk insufficiency, which were analyzed using an iterative process with NVivo12 software. Researchers focused on themes emerging from perceptions and reasons given by healthcare workers for human milk insufficiency.

**Results:**

Inability to produce adequate breastmilk was identified as a prevalent obstacle mothers face in the early postpartum period in both district and tertiary facilities in Malawi. The main reasons given by participants for human milk insufficiency were mothers’ perceived normalcy of milk insufficiency, maternal stress, maternal malnutrition, and traditional beliefs around food and eating. Three focused solutions were offered by participants to improve mother’s milk production – improving education for mothers and training for healthcare providers on interventions to improve mother’s milk production, increasing breastfeeding frequency, and ensuring adequate maternal nutrition pre- and post-partum.

**Conclusion:**

Health care workers perspectives shed light on the complexity of causes and solutions for human milk insufficiency in Malawi. This research highlights that a respectful professional relationship between health care workers and mothers is an essential bridge to improving communication, detecting human milk insufficiency early, and implementing appropriate interventions. The results of this study may help to inform research, clinical practice, and education in Malawi to improve human milk production.

## Background

The insufficient production of breastmilk is a substantial impediment to successfully implementing appropriate breastfeeding practices, including early and exclusive breastfeeding (EBF) (WHO, 2020). Inadequate milk production is one of the main reasons mothers give for weaning or using alternative methods like formula feed [1–3]. Human milk insufficiency (HMI) can lead to hypoglycemia, hypernatremia, nutritional deficiencies, and failure to thrive in newborns and infants [4]. HMI is not clearly defined in the literature, but it can be measured by volumetric milk outputs, infant weight gain, glucose levels, stool and urine output, muscle tone, and alertness [2]. Perceived insufficient milk (PIM) supply is well-defined in the literature as the state in which a mother perceives that she has an inadequate supply of milk to meet an infant’s needs [5]. Insufficient milk supply is a prevalent concern in 60–90% of women in low- and middle-income countries (LMICs) [6, 7]. Mothers with lower income and access to resources are at risk for higher levels of maternal stress and HMI than mothers with high income [8]. The concern for inadequate human milk production is warranted in Malawi, where there has been a decrease in both early initiation of breastfeeding and EBF in the first 6 months postpartum [1]. Additionally, Malawi has a high pre-term birth rate, at 10.1% [9] and prematurity increases the likelihood of breastmilk insufficiency, since weak suction pressures and immature sleep-wake regulation is associated with insufficient and delayed lactation [10].

While many studies highlight the physiological risks [4, 10] and maternal perceptions [6, 11, 12] which can influence HMI, very few examine the perspectives of health care workers (HCWs) and the reasons they give for inadequate milk production. HCW perspectives may provide critical information to understand and effectively remedy the challenge many mothers face with insufficient milk supply. Studies reporting on healthcare professional’s perspectives in providing breastfeeding support highlight inconsistencies between HCW perceived support and behaviours, uncertainty and confusion toward interventions to recommend, and gaps in practical knowledge and training skills [13, 14]. Investigators highlight a critical need for research to address the breastfeeding education and training needs of healthcare providers in different contexts, including through their own perspectives [14–16]. Furthermore, the etiology of inadequate human milk production is multifactorial and complex, and a comprehensive analysis of HCWs perceptions on HMI in LMICs is absent from the literature. The objective of this study is to understand HCW perspectives regarding inadequate human milk production in Malawi.

## Methods

### Research design

A secondary, retrospective analysis was conducted on 39 semi-structured, in-depth interviews with health workers on their perceptions and experiences with breastfeeding at hospitals in southern Malawi. We directed an investigative, cross-sectional, qualitative study to examine the perspectives on barriers and facilitators to implementing breastfeeding practices. This study is part of the larger project, “Integrating a neonatal healthcare package for Malawi” which aims to enhance the implementation of suitable cost-effective innovations to improve infant care at low-resource health facilities. A segment of the Innovating for Maternal and Child Health in Africa (IMCHA) project, this study received ethics approval from the University of Malawi College of Medicine (P.08/15/1783) and the University of British Columbia (H15–01463-A003).

### Sample

The interviews were conducted at a tertiary-level central hospital and three secondary-level district hospitals in southern Malawi from May to August 2019. The sample of participants was purposively drawn to include healthcare providers and supervisors working in maternal and neonatal health at the facilities [17].

### Data collection

Interviews with participants took place between May and August 2019 with a semi-structured topic guide. Interviews were conducted by four research nurses and a public health specialist who underwent a three-day intensive training in qualitative research methods led by ALMN and MWK. Data collectors introduced themselves as conducting research to understand barriers and facilitators to implementing breastfeeding support interventions. Face-to-face, 30–60-min interviews were conducted within the facilities. Participants provided written informed consent and were required to fill out a demographic information form. Interviews were conducted in English or *Chichewa*, the major local language in Malawi. We obtained written consent from each participant before any study procedures [17].

### Data analysis

This study used a combined conventional and directed content analysis applying an iteration process [18, 19]. This combined approach incorporated inductive and deductive coding to synthesize apriori issues elucidated from existing literature with novel themes that emerged from the data. Researchers generated initial codes based on the information from the literature review process, and an inclusive coding framework was distinguished after researchers familiarized themselves with interview transcripts. NVivo 12 software (QSR International, Melbourne, Australia) was used for multiple rounds of coding interview transcripts in a constant loop process. Predetermined and emerging themes were indexed into similar, recurring, or nuanced codes, and subcategories within each theme were examined. Themes were continuously refined and linked to existing literature.

## Results

**Fig. 1:**
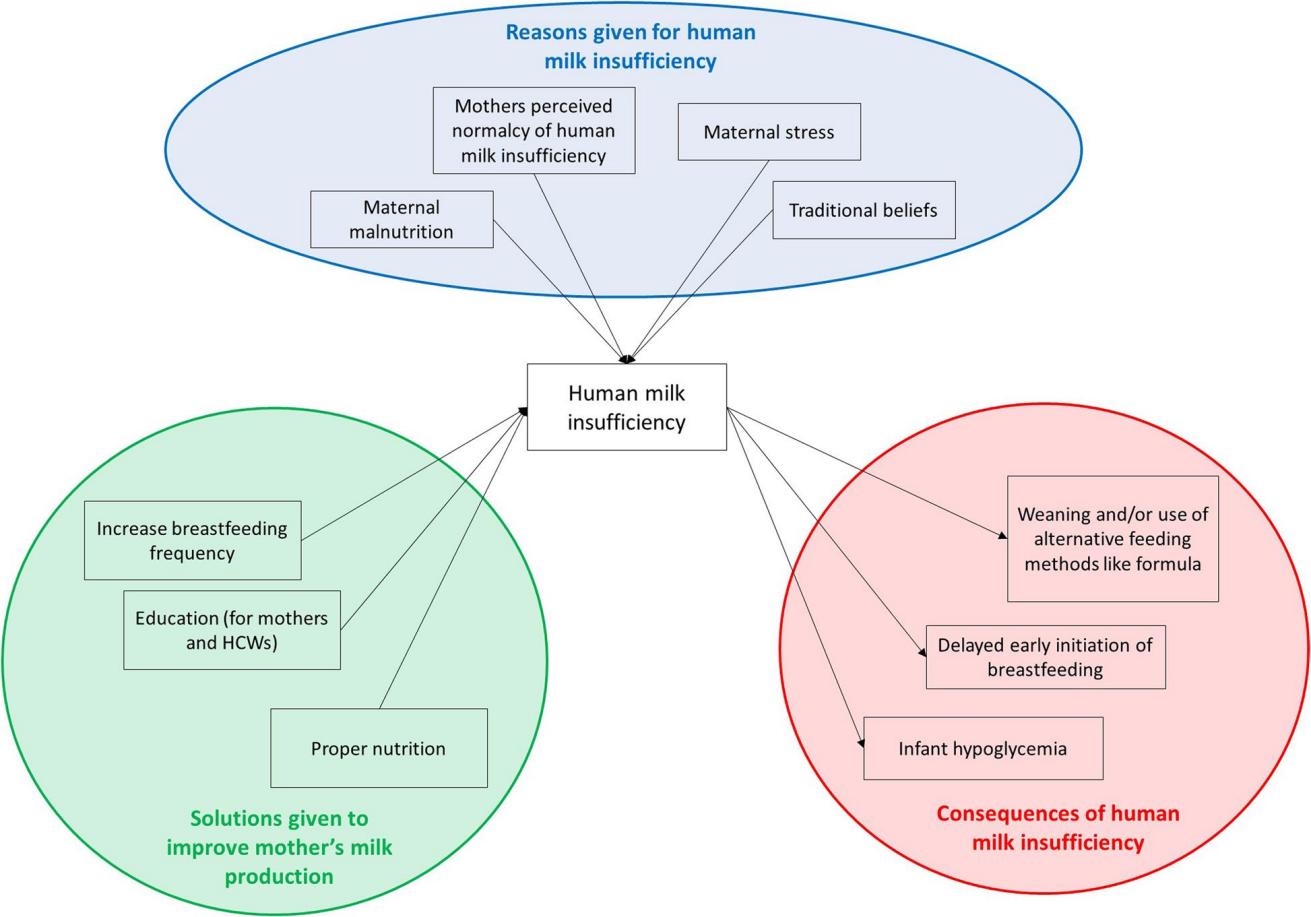
Connectivity of reasons given for inadequate human milk production, consequences of human milk insufficiency, and solutions given to improve mothers’ milk production, based on reporting from health care workers from district and tertiary hospitals in Malawi. HCW = health care worker

Interviews were conducted with 39 health care workers, of which 11 were nurses. Other HCWs interviews included Midwives, Clinical Officers, and Medical Doctors. We have organized the information shared by HCWs into three main categories: the problem of milk production, the reasons given for insufficient breastmilk production, and potential solutions that were shared to improve breastmilk production. These three main themes are detailed with direct quotations from respondents, and the reasons and potential solutions for breastmilk production are broken down into specific sub-categories. The flow of results is further illustrated in Fig. 1.

### The problem of insufficient human milk production

According to HCWs, inability to produce enough breastmilk was stated as the most prevalent obstacle to breastfeeding that mothers faced. Participants stated that mothers had difficulties producing milk in the first week postpartum. A district hospital clinical officer mentioned that “… most mothers have difficulties to produce milk on the first day or second day of life of the baby”. A district hospital nurse stated, “The main challenge is inadequate milk production, that’s the main challenge that we normally face, especially these young mothers with first born child so that’s the main challenge”. Some HCWs did not specify whether they were reporting measured or perceived breastmilk insufficiency.

HCWs regarded milk insufficiency as problematic and identified three main consequences associated with HMI, including delayed early initiation of breastfeeding, premature weaning and/or the use of alternative feeding methods, and infant hypoglycemia (Fig. 1). The most stated consequence was delayed initiation of breastfeeding or infrequent feeding, which can lead to hypoglycemia. Hypoglycemia refers to a low blood glucose concentration, which can increase neurodevelopmental disabilities if left untreated [20]. A nurse matron at a district hospital stated, “This may affect the mother because milk production may delay to come. It may also affect the baby because when the baby is not getting enough milk the baby may develop some problems such as hypoglycemia”. Participants also mentioned that infants who were not consuming enough breastmilk were more prone to infections and dehydration.

“The baby is over a week is not admitted [at the hospital]. So that baby came with convulsions. When we were asking parents were quiet, so we said what is happening, when we checked the sugar, it was very low and the baby was very sick that’s when we discovered that the baby was not breastfeeding because milk was not coming out. I am not saying that was the only one there are many cases.” Tertiary hospital Pediatric specialist

### Reasons given for inadequate human milk production

Five reasons were given for inadequate breastmilk production: its perceived normalcy among mothers, maternal stress, maternal malnutrition, and traditional beliefs around food and eating (Fig. 1).

#### Perceived normalcy

Insufficient milk production within the first few days after birth was reported to be common among mothers and perceived to be normal among mothers. HCWs indicated that many mothers believed that if they did not produce milk immediately after birth, it would be produced in time and there was no urgency or alarm. Because mothers perceived early milk insufficiency as normal, they did not inform the HCWs of their milk deficit. Nurses shared that low educational levels of some mothers, particularly in the rural districts, hindered capacity to understand breastfeeding counselling and their comfort to ask questions or approach HCWs with problems. For example, a district hospital nurse mentioned “… Most of our women here are, their literacy levels are very low … Most of the times they don’t even understand … even if they have questions they are always scared [and] they can’t ask”.

“[T] he perceptions they have is that they think that when breastmilk is not coming soon after delivery, they should just wait for it to start on its own, which is false, they need to stimulate it, when they put the baby on the breast the baby stimulates the breast so that the hormones, so that the milk starts coming out.” Tertiary hospital nurse

“The misunderstanding is due to difficulty in comprehending the process that can trigger the milk to come out. For instance, if they put the baby on their breast the message goes to their brain which commands the responsible gland to release the hormone responsible for producing milk. The main reason is what we call knowledge deficit especially among those who come from remote areas. They need special counseling that they should continue putting the baby to their breast to command the hormone to produce milk.” Tertiary hospital nurse

#### Maternal stress

HCWs stated that mothers who experienced stress had difficulties breastfeeding because they were less likely to produce sufficient amounts of breastmilk, compared to women who were less stressed. Family stress, gender-based violence, and many household responsibilities were stated to cause stress, which could interfere with mothers’ ability to produce enough milk. HCWs affirmed that maternal well-being and relaxation were crucial for adequate production of milk.

“If the woman is failing to breastfeed and because of the resulting stress the milk production continues to be a problem, and also when people from home come and tell the mother to give the baby milk because they feel it is being dehydrated.” District hospital nurse

“When the woman is psychologically prepared and when she has learnt properly about lactation support and also if her family is on peace with no stress, because that would make the husband to easily support the mother, but with family having misunderstanding it is usually hard for the mother to produce milk or to control the baby.” District hospital nurse

“If they have many babies and many things to do, family problems, gender based violence, because it’s all about psychology, the way the woman is feeling, one with clear mind and one with problems they differ when it comes to breast feeding, the one with problems may not even be able to produce milk and many other responsibilities may make the mother not adequately breast feed the baby.” District hospital nursing officer

#### Malnutrition

HCWs reported a connection between poor maternal nutrition and inadequate production of breastmilk. Participants stated that maternal willingness to breastfeed and an infants’ ability to properly suck was futile for breastfeeding if the mother was malnourished. District hospital clinical officers stated “… Breastfeeding needs nutritious foods, if there is no nutritious food, don’t expect much milk” and “… The mother may be willing to breastfeed, but if the mother has poor nutrition status, it’s impossible. The baby may be willing to suck, but if the milk is not coming out, it’s impossible”. The responses from HCWs do not specify whether they were referring to perceived or actual breastmilk insufficiency.

#### Traditional beliefs

Many participants stated that traditional beliefs around breastfeeding have progressed, specifically in the acceptance of colostrum. However, some indicated the presence of traditional beliefs about certain foods, which may affect breastmilk production. For example, some believed that eating cassava or roasted maize after childbirth would ensure adequate production of mother’s milk, suggesting that mothers may think that insufficient milk production may be linked to not eating these food items first.

“I have heard that they should eat roasted maize, they should eat cassava, so that the milk should start coming out … . Let’s say the mother who has done caesarian section, it would take the mother maybe 12 hours before taking anything like food, so it means if they are waiting for them to eat cassava first so that they should breastfeed, it means the baby can easily die of hypoglycemia...” Tertiary hospital nurse

“The other thing is they should be eating high nutritious food, so that they should be able to produce milk … For example, we encourage women to be eating eggs, so some of the traditional beliefs inhibit that, so it is a challenge.” District hospital Clinical officer

### Solutions given to improve human milk production

Three focused solutions were offered to improve mother’s ability to produce sufficient milk, including increasing the frequency of breastfeeding, improving maternal education on lactation and breastfeeding as well as HCW training on interventions to improve mother’s milk production, and ensuring mothers have access to adequate nutrition while breastfeeding (Fig. 1). However, a proportion of HCWs reported feeling uncertain on how to best support mothers experiencing HMI (see discussion on *Ambiguity below*).

#### Increase breastfeeding frequency

HCWs shared successes in advising mothers to increase feeding frequency to improve the production of human milk. A common understanding amongst HCWs was that mothers produce more milk the more frequently they breastfeed. HCWs did not indicate if or how they assessed mothers milk production, so it is unclear whether they are referring to perceived or actual milk production.

“There was a time where the woman wasn’t able to produce milk, and we told her that to be putting the baby frequently on the breasts, she did that for the whole day because she was understood, and then later she started producing milk and the baby breastfed.” District hospital nurse

“The woman was not producing milk, so we agreed that we should keep on encouraging the mother to keep on attempting on putting the baby on the breasts on every 15 minutes so that maybe the milk would come out.” District hospital nurse

#### Education

HCWs indicated they would like to learn how to better support mothers who are not able to produce enough milk. A pediatric specialist at the tertiary hospital stated, “I think more of the practical skills; things like how to help women who are having problems of lactation, how to help them increase milk production”. HCWs reported feeling overwhelmed and confused about what solutions to recommend for mothers experiencing HMI. There was some uncertainty among HCWs on whether they should promote the use of formula milk, expressed human milk, or encourage mothers to increase the frequency of breastfeeding. These narratives highlight a potential gap in training of HCWs in this area.

“I would like to learn more how we can help mothers who can’t produce milk. How can we help them because this is a problem that some mothers are not able to produce breast milk, and we have to know the best way we can help these mothers.” District hospital Clinical Officer

In addition to improved HCW training, increasing education for mothers on proper positioning and latching, feeding frequency, and the lactation process were also suggested to improve breastmilk production.

“There was a woman that was finding lactating or holding the baby difficult … but when we explained … how she can position and hold the baby when breastfeeding, every complication [that] brought about [a] lack of breastfeeding … got back to normal. The milk production increased, which stopped dehydration and the crying stopped...” District hospital nurse

“The misunderstanding is due to difficult [y] in comprehending the process that can trigger the milk to come out. For instance, if they put the baby on their breast, the message goes to their brain which commands the responsible gland to release the hormone responsible for producing milk. The main reason is what we call knowledge deficit especially among those who come from remote areas to understand that sooner or later milk will come out. They need special counseling that they should continue putting the baby to their breast to command the hormone to produce milk.” Tertiary hospital nurse

#### Proper nutrition

HCW participants from district hospitals indicated that many mothers experienced poverty, and this resulted in poor maternal nutrition, which impeded their ability to produce enough milk. Consequently, hospital nutritional support was recommended. These results were not indicated in the interviews with HCWs from tertiary hospitals.

“… most of the patients are poor so I think it’s necessary to bring in some mechanisms that will help them to buy basic needs that will support them. As you know breast feeding needs nutritious foods; if there is no nutritious food, don’t expect much milk.” District hospital Clinical officer

#### Ambiguity

Some HCWs reported feeling pressured by families to help improve mother’s milk production but uncertain about what method(s) to use, and whether it would be appropriate to recommend formula feed when mothers reported experiencing HMI. The ambiguity reported by HCWs reveals potential gaps in established best practices for supporting mothers with HMI in these Malawian health facilities.

“Then you try every means possible for lactation to be established, but it’s not helping, the milk can’t come out for days, even a day or two days, then there will be pressure from guardians to say should we use lactogen or something, you know that’s not recommended, but you are still at pressure to say how then do I make this milk come, how do I establish it, so that has been a major problem I don’t know how we can look into it, but there are circumstances, that happens anyway.” District hospital nurse

“To me that’s a challenge, and you find that some families … some mothers, when they see that day one is gone, the milk is not coming out, the next day they bring a tin of milk and they will say ‘midwife since yesterday milk is not coming out though I am eating, so we have decided we buy the [formula] milk’, while the baby will still be trying to suck from the mother … So in that situation we become in dilemma … do we start this baby on the formula milk[?] [W] ill she continue [breastfeeding] or not?” Tertiary hospital nurse

## Discussion

### Summary of primary findings

The purpose of this research was to examine Malawian HCWs perceptions on lactation and insufficient breastmilk production in the first few weeks postpartum. The three main themes derived from interviews with HCWs included the problem of insufficient breastmilk production, the reasons given for HMI, and solutions to improve HMI. Breastmilk insufficiency was described as a main challenge mothers faced to implementing breastfeeding after birth, yet many HCWs did not distinguish perceived versus actual breastmilk production in their responses. The reasons reported for milk insufficiency included mothers’ perceived normalcy, maternal stress, maternal malnutrition, and traditional beliefs. While some HCWs were uncertain on how to support mothers experiencing HMI in the first few weeks postpartum, potential solutions given by other HCWs included increasing breastfeeding frequency, educating mothers and improving training for HCWs, and supporting maternal nutrition at the hospital. The intersection of reported reasons, solutions, and consequences of HMI as described by HCWs at Malawian healthcare facilities can be seen in Fig. 1.

### Existing literature on the topic & what this study adds

Existing literature on HMI highlights maternal and infant physiological factors that can lead to the insufficient production of mother’s milk. Kent and colleagues highlight infant functional reasons for insufficient milk supply, including medical conditions like preterm birth, hypothyroidism, congenital heart disease, cleft palate, and neurological disturbances [2]. Maternal factors that may lead to HMI include inadequate mammary tissue, disturbed ductal and neurological pathways, and abnormal concentrations of hormones including oestrogen, prolactin, progesterone, oxytocin, growth hormone, glucocorticoids, and insulin [21]. Emerging research on inadequate breastmilk production is more focused on the psychological explanations. High levels of maternal stress have been tied to decreased production of human milk due to the inhibition of the milk ejection reflex [22]. Notably, mother’s *perception* of insufficient milk production is the most common cause of breastfeeding discontinuation, defined as perceived insufficient milk (PIM) [11, 12, 23]. Maternal PIM commonly leads to infrequent suckling and reduced mammary tissue stimulation, which often results in a true reduction in mother’s milk production [2, 5, 9, 22]. Solutions for milk insufficiency discussed in the literature highlight relaxation techniques for stressed mothers [22], adequately draining mother’s milk between feeds [2], increasing feeding frequency [2, 3], and using galactagogues [2, 3, 22]. Existing literature indicates that early initiation of breastfeeding is directly proportional to early milk production and correlates with adequate milk production during established lactation [3]. This suggests that interventions to promote early initiation of breastfeeding in the first few days postpartum are critical to preventing HMI. This suggestion is consistent with the recommended solution from HCWs to increase breastfeeding frequency in early stages after birth as a way of improving mother’s milk supply. Furthermore, HCW perspectives surrounding perceived and actual milk insufficiency seem to be absent from the current literature. HCW perspectives presented in this study give valuable insights into the postpartum supports (or lack thereof) that are available to breastfeeding mothers in the first few weeks postpartum and how they may impact breastmilk production thereafter. These ‘birds-eye-view’ observations provide critical intel for practice and future research on this topic.

### Implications for practice & future research

Results from our study emphasize that milk insufficiency was perceived to be normal among caregivers, which may be the result of its prevalence in Malawi and the widespread misunderstanding of the lactation process. Low maternal educational levels – especially in rural districts – may challenge mothers’ capacity to understand breastfeeding counselling, particularly if nurses describe the process of lactation in technical, hormonal terms. Low maternal educational levels and poor understanding may also impact caregiver comfort to approach HCWs with questions, and gaps in communication can exacerbate challenges. Maternal illiteracy has been linked with poorer feeding practices and chronic undernutrition among pregnant women and their infants in Malawi [24] and Uganda [25]. Low literacy levels are often an indicator of poverty [12], and mothers who are impoverished are often unable to achieve proper nutrition and are not likely to produce enough human milk; as was reported by Clinical Officers at the district hospitals. Findings from our study suggest that future research is needed to explore the intersection of poverty, low literacy levels, the perception of normalcy toward breastmilk insufficiency, and actual HMI in breastfeeding mothers.

HCWs from both district and tertiary facilities highlighted uncertainty in their recommendations for mothers with HMI, including when lactation support should be provided, whether they should advocate for increased feeding frequency, or alternative feeding supplements like infant formula. In Malawi, Nurse-midwives provide lactation support to women during antenatal care education sessions [26], but this information is minimally repeated during and after delivery [17]. This is critical and consistent with our findings, since many HCWs suggested a need for improvements in education and training, such that they may better support mothers with lactation issues in the early postpartum period. Since HCW perspectives are not often targeted, this is a novel contribution to the literature on breastfeeding support. Reports of ambiguity by HCWs highlight a potential gap in education and training for HCWs in Malawi, and many HCWs stated they would like to learn more about how to support mothers with low milk production. Methods to support mothers include increasing breastfeeding frequency to support adequate mammary tissue stimulation (from the infant’s sucking stimulus), proper drainage of breasts, and maintaining appropriate concentrations of hormones [10].

Many HCWs discuss their experience with mothers who are unable to produce breastmilk, yet they do not differentiate whether the determination of milk insufficiency was perceived or actually measured (i.e. milk volume, glucose levels, infant weight gain). In studies examining PIM, mothers and HCWs used infant satiety cues as their main indication of milk supply, which are not necessarily accurate measures [2, 23, 27]. Additionally, clinical cues such as latching, sucking patterns, changes in breast fullness, and noticeable swallowing are unreliable indicators of human milk amount [2, 26]. Furthermore, monitoring and quantifying mother’s milk supply or interval infant weighing growth curves in the early postpartum period are potential, cost-effective solutions to identify mothers in need of further lactation support [3]. These findings highlight a need for improved education and training for HCWs on practices to identify and support mothers with insufficient milk production. Now that we have a better understanding of HCW perspectives on milk insufficiency and their role in providing support to mothers, future research is recommended to explore mother’s perceptions of HCW support when they are experiencing milk insufficiency as well as to tease out the potential differences between actual and perceived milk insufficiency.

### Limitations

A significant limitation of this secondary, retrospective analysis was the inability to distinguish between actual HMI and PIM in HCW’s responses. While HMI and PIM may be correlated in some instances, if a mother has PIM, actual milk production should be measured before initiating any intervention [2]. While the ambiguity surrounding HMI and PIM in HCW responses was a limitation of this study, it also provided insights regarding areas of clinical practice that require further research and potential instruction in Malawian healthcare facilities. Having said that, a limitation of this study was that it focused on perceptions HCWs but did not examine beyond the community level or fully investigate health system constraints which may influence current practices.

## Conclusion

The most prominent reason HCWs provided for the insufficient production of human milk was mother’s perceived normalcy, which highlighted inadequate communication between HCWs and mothers. Many HCWs reported uncertainty on what solutions to recommend for mothers experiencing HMI and stated that they would like to learn more practical skills to assist mothers experiencing problems producing enough milk, highlighting a gap in current medical education provided to HCWs in Malawi. The findings from this study may be helpful in guiding the development of effective interventions in clinical and community settings for HMI. HCWs can then provide these interventions to improve breastfeeding practices for mothers in LMICs to improve infant health outcomes.

## Data Availability

The datasets used and/or analysed during the current study are available from the corresponding author on reasonable request.

## Abbreviations

EBF: Exclusive breastfeeding
HCW: Health care worker
HMI: Human milk insufficiency
IMCHA: Innovating for Maternal and Child Health in Africa
LMIC: Low- and middle-income country
PIM: Perceived insufficient milk
